# Unsupervised Eye Pupil Localization through Differential Geometry and Local Self-Similarity Matching

**DOI:** 10.1371/journal.pone.0102829

**Published:** 2014-08-14

**Authors:** Marco Leo, Dario Cazzato, Tommaso De Marco, Cosimo Distante

**Affiliations:** 1 National Research Council of Italy, Institute of Optics, Arnesano, Lecce, Italy; 2 Faculty of Engineering, University of Salento, Lecce, Italy; Save Sight Institute, Australia

## Abstract

The automatic detection and tracking of human eyes and, in particular, the precise localization of their centers (pupils), is a widely debated topic in the international scientific community. In fact, the extracted information can be effectively used in a large number of applications ranging from advanced interfaces to biometrics and including also the estimation of the gaze direction, the control of human attention and the early screening of neurological pathologies. Independently of the application domain, the detection and tracking of the eye centers are, currently, performed mainly using invasive devices. Cheaper and more versatile systems have been only recently introduced: they make use of image processing techniques working on periocular patches which can be specifically acquired or preliminarily cropped from facial images. In the latter cases the involved algorithms must work even in cases of non-ideal acquiring conditions (e.g in presence of noise, low spatial resolution, non-uniform lighting conditions, etc.) and without user's awareness (thus with possible variations of the eye in scale, rotation and/or translation). Getting satisfying results in pupils' localization in such a challenging operating conditions is still an open scientific topic in Computer Vision. Actually, the most performing solutions in the literature are, unfortunately, based on supervised machine learning algorithms which require initial sessions to set the working parameters and to train the embedded learning models of the eye: this way, experienced operators have to work on the system each time it is moved from an operational context to another. It follows that the use of unsupervised approaches is more and more desirable but, unfortunately, their performances are not still satisfactory and more investigations are required. To this end, this paper proposes a new unsupervised approach to automatically detect the center of the eye: its algorithmic core is a representation of the eye's shape that is obtained through a differential analysis of image intensities and the subsequent combination with the local variability of the appearance represented by self-similarity coefficients. The experimental evidence of the effectiveness of the method was demonstrated on challenging databases containing facial images. Moreover, its capabilities to accurately detect the centers of the eyes were also favourably compared with those of the leading state-of-the-art methods.

## Introduction

As one of the most salient features of the human face, the eyes and their movements play an important role in expressing a person's desires, needs, cognitive processes, emotional states, and interpersonal relations. For this reason the definition of a robust and non-intrusive system for the detection and tracking of the eyes is crucial for a large number of applications (e.g. advanced interfaces, control of the level of human attention, biometrics, gaze estimation, early screening of neurological pathologies).

A detailed review of recent techniques devoted to this topic can be found in [1] where it is clear that the most promising solutions use invasive devices (*Active Eye Localization Systems*). In particular, some of them are already available on the market and require the user to be equipped with a head mounted device [2] while others obtain accurate eye location through corneal reflection under active infrared (IR) illumination [3] [4]. These systems are generally expensive and not very versatile (sice they often require a preliminary calibration phase).

On the other hand, *Passive Eye Localization Systems* attempt to obtain information about the eyes' location just starting from images supplied from a monocular video stream: they explore the characteristics of the human eye to identify a set of distinctive features and/or to characterize the eye and its surroundings by the color distribution or filter responses. This way of proceeding introduces several challenges that each solver must address:

1. the iris is often partially occluded by eyelids, eyelashes, and shadows, especially for oriental users;
2. the iris can also be occluded by specular reflections when the user wears glasses;
3. the pupillary and limbic boundaries are non-circular and therefore can lead to inaccuracy if fitted with simple shape assumptions;
4. images can be affected by defocusing, motion blur, poor contrast, oversaturation, etc.

To address these challenges many advanced eye detection algorithms have been proposed in the last two decades. The method proposed by Asteriadis et al. [5] assigns a vector to every pixel in the edge map of the eye area, which points to the closest edge pixel. The length and the slope information of these vectors are consequently used to detect and localize the eyes by matching them with a training set. Timm and al. [6] proposed an approach for accurate and robust eye center localization by using image gradients. They derived an objective function whose maximum corresponds to the location where most gradient vectors intersect and thus to the eye center. A post-processing step is introduced to reduce wrong detection on structures such as hair, eyebrows or glasses. In [7] the center of (semi)circular patterns is inferred by using isophotes. In a more recent paper by the same authors, additional enhancements are proposed (using mean shift for density estimation and machine learning for classification) to overcome problems that arise in certain lighting conditions and occlusions from the eyelids [8]. A filter, inspired by the Fisher Linear Discriminant classifier and requiring a sophisticated training, is, instead, proposed in [9]. In [10] a cascaded AdaBoost framework is proposed. Two cascade classifiers in two directions are used: the first one is a cascade designed by bootstrapping the positive samples, and the second one, as the component classifiers of the first one, is cascaded by bootstrapping the negative samples. A similar approach is proposed in [11] where the Adaboost-cascade is coupled with a reflection removal method to exclude specularities in the input images. A method for precise eye localization that uses two Support Vector Machines trained on properly selected Haar wavelet coefficients is presented in [12]. In [13] an Active Appearance Model (or AAM) is used to model edge and corner features in order to localize eye regions whereas in [14] an ensemble of randomized regression trees is used. Also active boundary detection strategies can be used for this purpose [15] [16]: they can be used to evolve a contour that can fit also to a non-circular iris boundary. However, strategies to improve pupil and iris localization accuracy and to reduce their parameter sensitivity, are still under investigation [17].

Unfortunately, all the above methods use either a supervised training phase for modeling the appearance of the eye or ad-hoc reasonings to filter missing or incorrect detections of the eyes. For these reasons, although they achieved excellent performance in the specific contexts in which were tested, their use in different situations (especially in unconstrained environments) has to be preceded by some adjustments of the previously learned models. On the other hand, well known unsupervised approaches in this field are those proposed in [18] and [19], which find circular shapes by using the integro-differential operator and the Hough Transform respectively. However, their ability to find the eye relies on very simple and rigid model and, thus, they suffer the partial occlusions or deformations of the iris and their performances strongly degrade also in the case of noisy or low resolution images. An early tentative to introduce a more efficient pupil detection approach that does not require any training phase (or post filtering strategy) has been recently proposed in [20]. In that paper the classical Circular Hough Transform is biased by local appearance descriptors. Although the detection performances are encouraging, there is compelling evidence that the Hough transform limits the operability of the system due to both its high computational load and its inability to manage the discontinuities in the edges of the circular regions (generated by the presence of the eyelids and eyelash). This paper tries to overcome the aforementioned limitations by introducing a more accurate and computationally efficient strategy for the detection of the eyes' centers: it relies on the combination of the differential analysis of the image intensities and the local appearance variability represented by self-similarity coefficients. Experimental evidence of the effectiveness of the proposed solution was proven on challenging databases containing facial images of different subjects (also belonging to different ethnic groups) acquired under different lighting conditions and with different scales and poses. The rest of the paper is organized as follows: next section gives an overview of the proposed solution and then, in the related subsections, it details the three operating steps aimed at localizing the pupil. Then experimental proofs are described and discussed in the subsequent section and, finally, conclsions are reported in the last section of the paper.

## The Proposed Approach

**Figure 1:**
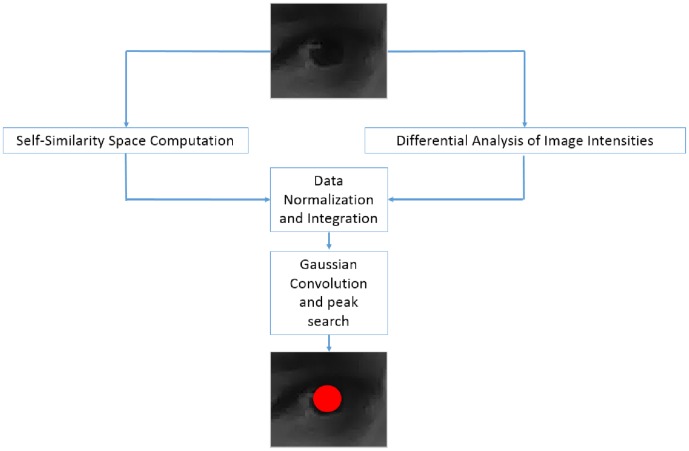
A schematic representation of the algorithmic procedures.

Similarly to the related works in the previous section, the proposed solution operates on periocular images which can be specifically acquired (this way a high resolution close-up view of the eye is generally available) or (eventually automatically) cropped from a large facial image. In figure 1 a schematic representation of the involved algorithmic procedures is shown. For each input image, on the one side the self-similarity scores are computed in each pixel and, on the other side, the differential analysis of the intensity levels is performed. The outcomes of these preliminary steps are then normalized and integrated in a joint representation where, after a smoothing with a Gaussian Kernel, the most circular and self-similar regions emerge. Finally the peak in the achieved data structure is found and it is assumed to correspond to the center of the eye. Next subsections will explain the implementation details of each procedural step.

## Self-Similarity Space Computation

**Figure 2:**
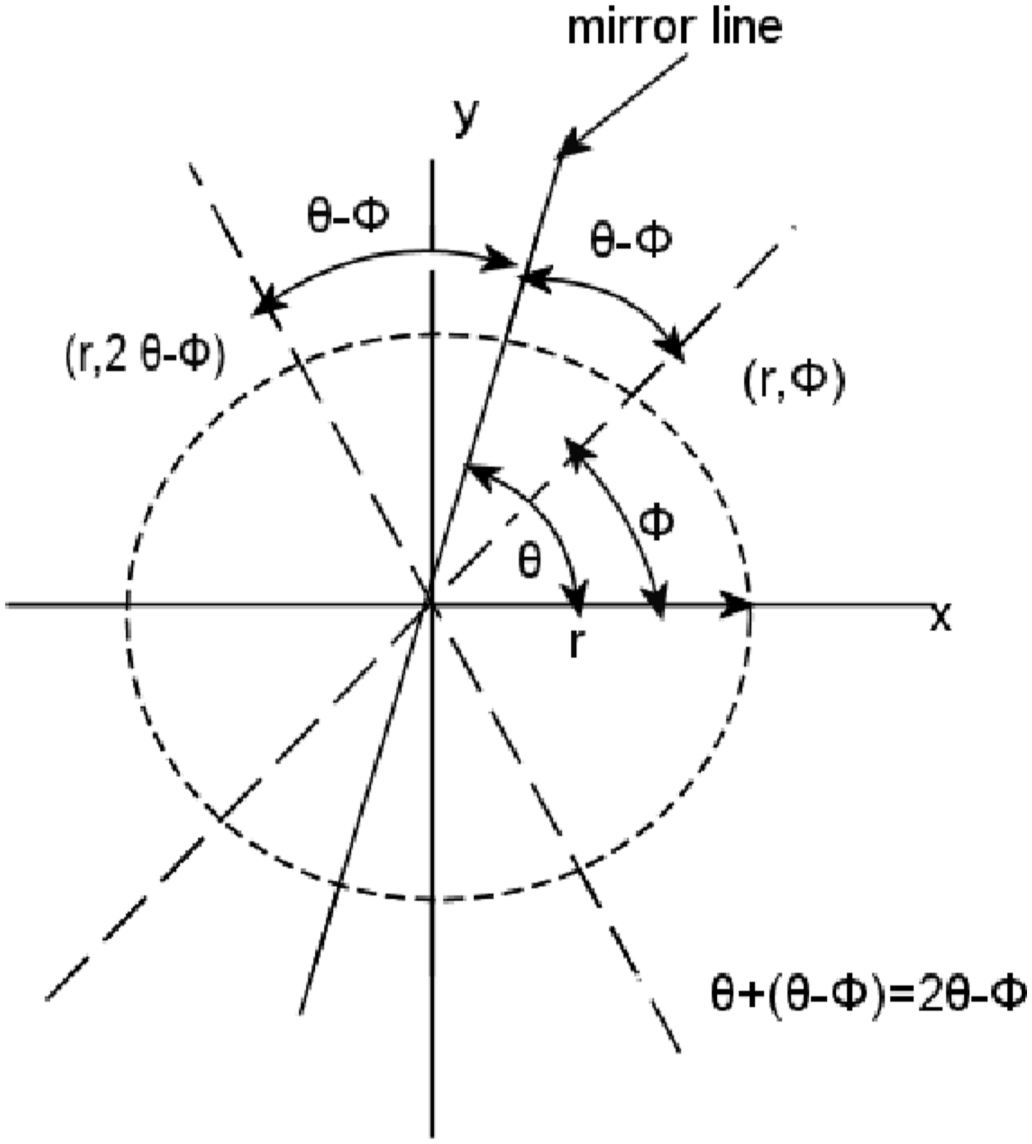
A reflection maps the location 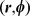 to location 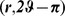.

The first computational step aims at searching for regions with high self-similarity, i.e. regions that retain their peculiar characteristics even under geometric transformations (such as rotations or reflections), changes of scale, viewpoint or lighting conditions and possibly also in the presence of noise. Self-similarity score can be effectively computed as a normalized correlation coefficient between the intensity values of a local region and the intensity values of the same geometrically transformed local region [21]. A local region is self-similar if a linear relationship exists, i.e.:
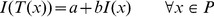
(1)where 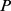 is a circular region of radius 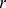 and 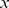 is a point located in 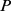. 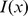 denotes the intensity value of the image 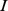 at location 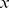, and 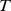 represents a geometric transformation defined on 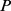. For the purposes of the paper, 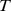 is limited to a reflection and a rotation. Both reflection and rotation preserve distances, angles, sizes, and shapes. To better clarify the notions of reflection and rotation into the specific context under consideration, point locations can be represented in polar coordinates, hence 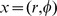. Every reflection is associated to a mirror line going through the center of 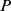 and having orientation denoted by 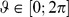. Having said that, a reflection is defined as the geometric transformation that maps the location 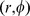 to location 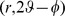 (see figure 2).

Similarly every rotation is defined by a centre and an angle. Let the centre of the rotation be the centre of 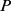 and let the rotation angle 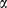 be one of the angles 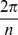, where 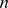 is a nonzero integer. A rotation maps the location 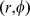 to location (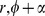).

Given these preliminary concepts, from the operational point of view, the cornerstone of this first phase is the search of the points that are closest to satisfy the condition in equation 1 considering that, on real data, it can hardly be fulfilled for all points of 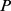. This way, highlighted points should correspond to the pixels of the eye which has both (almost) radial and rotational symmetry. In particular, the strength of the linear relationship in equation 1 can be measured by the normalized correlation coefficient:
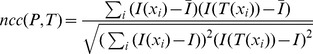
(2)


Here 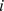 counts all points of 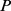 and 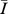 represents the average intensity value of points of 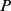.

At a given location, the normalized correlation coefficients in equation 2 can be computed for different mirror line orientations or different angles of rotation. All give information of region self-similarity.

In this paper the average normalized correlation coefficient computed over all orientations of the mirror line (*radial similarity map* 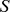) at a given location is used as a measure of region self-similarity. The self-similarity coefficients computed when 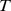 is a reflection are equal to those computed when 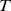 is a rotation. This has been mathematically proven in [21].

Let the sampling intervals for 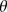 be 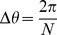, the similarity measure is then computed as
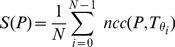
(3)


In order to cope with the analysis at different scales, this formula is computed for different radii 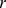 (i.e. the number of considered scales). This brings to the formulation of the equation for the computation of the multi-scale self-similarity:
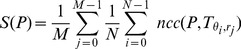
(4)where 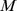 defines the sampling interval for 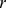, i.e. 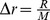.

To overcome the problems related to the processing near the borders of the input periocular image, the calculation of the self-similarity scores is performed only for those pixels belonging to a smaller region (i.e. discarding the outmost 10 pixels in each direction).

The self-similarity map 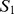 (of size 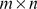) computed by equation 4 is the outcome of this first phase.

## Differential Analysis of Image Intensity

The second computational phase aims instead at the analysis of the geometric properties of periocular patches: this analysis is performed by introducing isophotes, i.e. curves connecting pixels in the image with equal intensity. Due to their intrinsic properties, isophotes are particularly suitable for objects detection and image segmentation: they follow constant intensity and therefore follow object shape both around edges as well as smooth surfaces. In particular, it has been demonstrated that their shapes are independent from rotation and varying lighting conditions, and, in general, isophote features result in better detection performance than intensities, gradients or Haar-like features [22]. Curvature 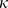 of an isophote, which is the reciprocal of the subtended radius 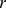, can be computed as:
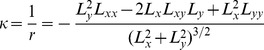
(5)where 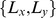 and 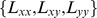 are the first- and second-order derivatives of the luminance function 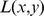 in the 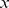 and 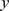 dimensions respectively (for further details refer to [23]).

Since the curvature is the reciprocal of the radius, equation 5 is reversed to obtain the radius of the circle. The orientation of the radius can be estimated by multiplying the gradient with the inverse of the isophote curvature. This way the displacement vectors to the estimated position of the centers can be computed as
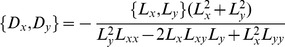
and then they can be mapped into an accumulator 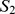 that is the outcome of this processing phase.

**Figure 3:**
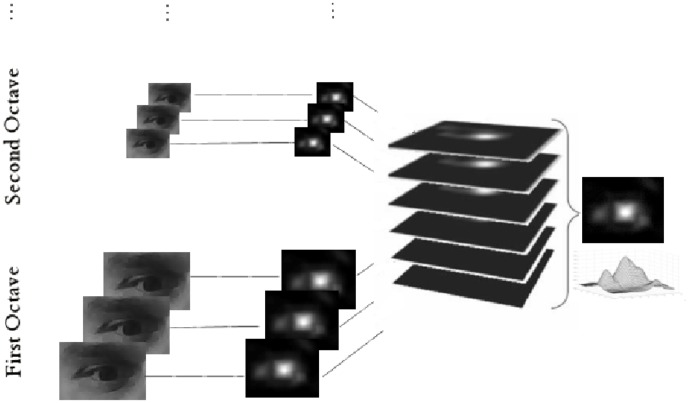
The scheme of the pyramidal analysis of the image intensity variations.

In order to face possible changing in scales a Difference of Gaussian Pyramid is generated and the above procedure is applied on each element of the pyramid. All the computed accumulation spaces are then linearly summed up into a single space that is the output of this computational step. This process is schematically represented in figure 3 and it is implemented according to [24].

## Pupil Localization

The final step of the proposed approach integrates the corresponding self-similarity and differential accumulator spaces. Both data structures are normalized in the range 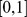 and then pointwise summed. The resulting accumulation space is then convolved with a Gaussian Kernel in order to allow the areas with highest average score (on a neighborhood defined by the sigma of the kernel) to excel over those having some occasional large value mainly due to some noise. Finally the peak in the smoothed data structure is selected as the center of the eye.

**Figure 4:**
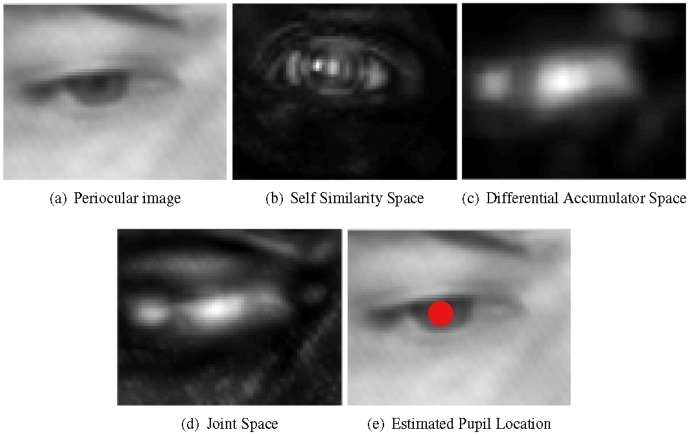
a) region containing a human eye; b) the corresponding accumulator space by Self-Similarity Analysis; c) the corresponding accumulator space derived from differential analysis of image intensity; d) smoothed joint space; e) pupil location.

Figure 4 shows an example of how the proposed procedure detects the pupil within a periocular image: subfigure 4(a) shows the cropped region of the eye whereas the corresponding numerical spaces built trough the self-similarity and differential analyses are shown in subfigures 4(b) and 4(c) respectively. Subfigure 4(d) shows instead the joint space obtained by point-wise adding self-similarity and differential accumulator spaces. Finally, subfigure 4(e) shows the estimated location of the pupil (i.e the peak in the joint space). Note how, in this joint representation, the area around the pupil is more emphasized than the representations in the individual spaces obtained through the analysis of the self-similarity and the differential analysis of the levels of intensity. In particular largest values (represented by the whitest pixels) are localized close to the pupil making its localization more accurate and robust to noise and changing in the imaging conditions. This will be extensively proven in the following section reporting experimental results.

## Experimental Results

Experimental evidence of the effectiveness of the method was achieved on challenging benchmark datasets containing facial images. We did not decide to use some of the datasets of periocular images (e.g. [25]) since, as already mentioned in the introduction section, they have been collected for biometrics purposes and then contain only close-up views of eyes acquired under well-controlled conditions of light, scale and pose (resulting from the active collaboration of the involved persons). Under these favourable operating conditions most of the methods are able to get very accurate results in the detection of the eye center and therefore it would not be possible to assess the real benefit of using the proposed approach. In contrast, the datasets of facial images are collected for a variety of purposes (surveillance, human-machine interaction, interactive gaming, etc..) and therefore the images in them are collected without specific constraints on the conditions of acquisition but rather, as we will see below, introduce some deliberately extreme operating conditions in order to allow an exhaustive test of the algorithms. Working on facial images, during the experimental phases, was thus necessary to introduce a preliminary face detection step to allow a quick extraction of the corresponding periocular patches. Any of the face detectors in the huge literature could be used to accomplish this additional task. For practical reasons (largely tested code is available on line), in the experimental phase the boosted cascade face detector proposed by Viola and Jones [26] was used. In particular the code (with default parameters) available with the Computer Vision System Toolbox of the MATLAB (R2012a version) was used and, once the face was detected, the periocular patches were then cropped using anthropometric relations. The cropped patches started from 20
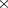
30 percent (left eye) and 60
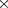
30 percent (right eye) of the detected face region, with dimensions of 25
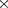
20 percent of the latter.

In the first experimental phase the BioID database [27] was used for testing and, in particular, the accuracy of the approach in the localization of the pupils was evaluated. The BioID database consists of 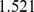 gray-scale images of 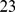 different subjects taken in different locations, at different times of the day and under uncontrolled lighting conditions. Besides non-uniform changes in illumination, the positions of the subjects change both in scale and pose. Furthermore, in several examples of the database, the subjects are wearing glasses. In some instances the eyes are partially closed, turned away from the camera, or completely hidden by strong highlights on the glasses. Due to these conditions, the BioID database is considered one of the most difficult and realistic database of facial images. The size of each image is 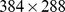 pixels and a ground truth of the left and right eye centers is provided with the database. The *normalized error*, indicating the error obtained by the worse eye estimation, is adopted as an accuracy measure of the eye locations. This measure is defined in [28] as
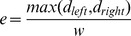
(6)where 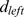 and 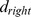 are the Euclidean distances between the estimated left and right eye centers and the ones in the ground truth and 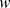 is the Euclidean distance between the eyes in the ground truth. In this measure, 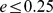 (a quarter of the interocular distance) roughly corresponds to the distance between the eye center and the eye corners, 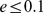 corresponds to the range of the iris and 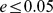 corresponds to the range of the pupil.

**Figure 5:**
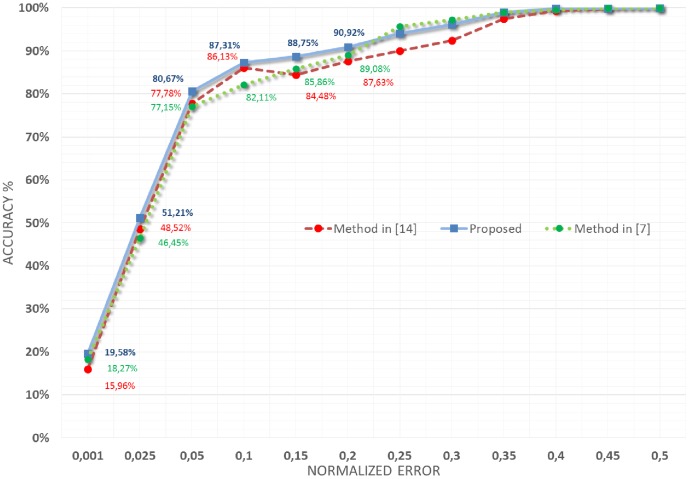
Results obtained on the BioID database and their comparison with those obtained using the strategy proposed in [20] and in [7].

In figure 5 the accuracy of the proposed approach on the BioID database is reported (continuous blue line). In particular, the y-axis reports the accuracy, i.e. the percentage of images in the database on which the pupils were localized with an error less than the normalized error (computed as indicated in equation 6) indicated by the corresponding value on the x-axis. The same figure reports also the pupil localization performances obtained on the same database by using the approach recently proposed in [20] (dashed red line) and in [7] (dotted green line).

As evident, the proposed approach significantly increased the performances in accuracy of the localization of pupils: in particular, considering the capability to remain into the actual pupil range (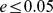), the performances increased from 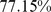 and 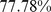 to 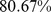 and, considering the localization into the iris range (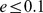), the performances increased instead from 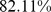 and 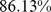 to 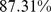.

**Figure 6:**
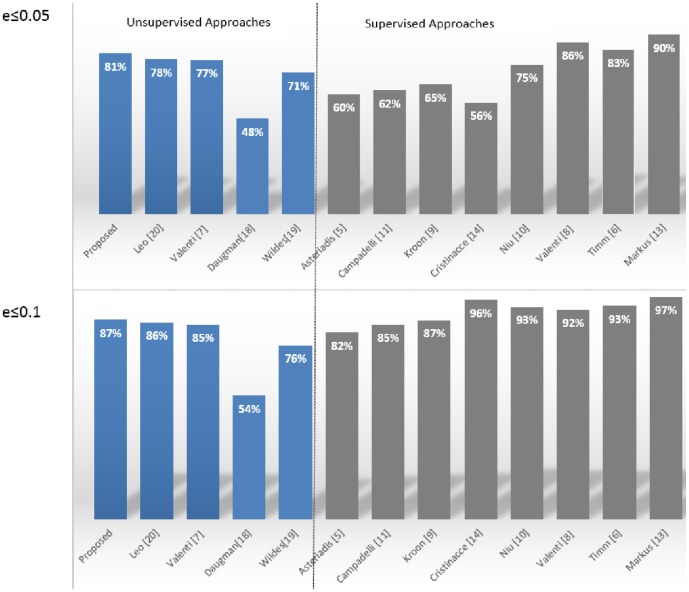
Comparison with state-of-the-art methods in the literature on the BioID database.

These results are very encouraging, especially in light of their correlation with those obtained by other leading state-of-the-art methods in the literature. To this end, in figure 6, the comparison (for normalized errors 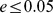 and 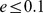) with the most accurate techniques (both supervisioned and unsupervisioned) in the literature is reported. Looking at the figure it can be seen that the proposed approach provided outstanding results considering that it outperformed most of the related methods, even some of them which use supervised training or post processing adjustments. In particular only the supervised methods proposed in [8], [6] and [14] provided better results both for 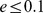 and 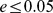 measures. These top-rated methods, however, utilize some learning procedures based on an accurate selection of training examples and/or a specific post-processing arrangements for filtering incorrect detections: this way the excellent performance exhibited on the BioID database cannot be replicated in different operating contexts without some adjustment of the working parameters and/or of the elements in the training set. In particular the method in [14] uses a machine learning algorithm (named randomized regression tree) to discover eye features, [6] adds a priori knowledge and selected thresholds to filter wrong detections and finally [8] introduces a feature-space analysis (mean shift) and machine learning techniques to validate the estimated eye centers. From the figure it is also evident that classical unsupervised approaches ([18] and [19]) failed to detect the center of the eye due to the uncontrolled acquisition, occlusions of the iris/pupil boundaries (due to eyelids and eyelashes) and reflections. The aforementioned unsupervised approaches are indeed based only on the difference in pixel intensity between internal and external region of the iris and thus they can fail if this difference becomes smoother as happens in the considered facial images.

**Figure 7:**
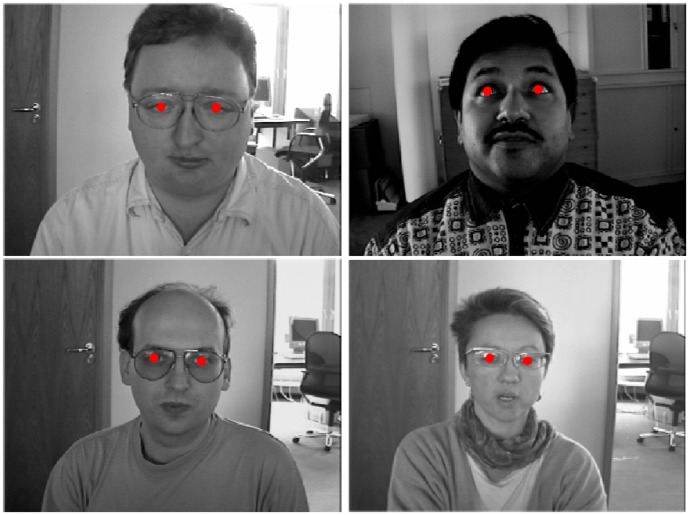
Some images of the BioID database in which the approach correctly detected the pupils. Reprinted from [27] under a CC BY license, with permission from Ho B. Chang, original copyright 2001.

In figure 7 some images of the BioID database in which the proposed approach correctly located the pupil in both eyes are shown even if they were acquired in challenging conditions: in fact, in three of them, people wore glasses and in the remaining ones the eyes were half-closed or gaze was turned away from the camera.

**Figure 8:**
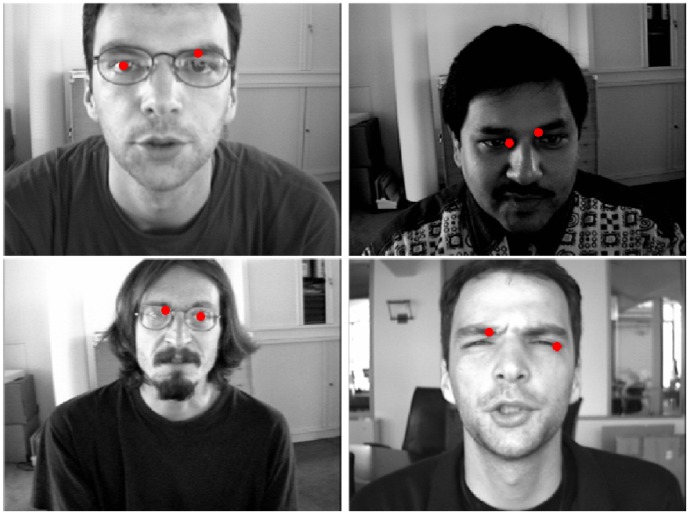
Some images of the BioID database in which the approach failed in the detection of the pupils. Reprinted from [27] under a CC BY license, with permission from Ho B. Chang, original copyright 2001.

Figure 8 reports, instead, some images of the database in which the approach failed in the detection of the pupils of one or both eyes. In most cases, the errors were due to very strong highlights on the glasses. Sometimes, due to particular head poses, the system localized the pupil on the eyebrows.

To systematically evaluate the robustness of the proposed pupil locator to lighting and pose changes, one subset of the Extended Yale Face Database B [29] was then used in the second experimental phase. The full database contains 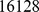 images of 28 human subjects under 9 poses and 64 illumination conditions. The size of each image is 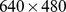 pixels. In particular, the proposed solution was tested on the 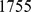 images belonging to the subsets 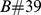, 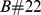 and 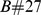. This choice was useful also to verify the sensitivity of the system to different ethnic groups. The performance in accuracy of the proposed approach on this second challenging dataset are reported in table 1.

**Table 1:** Accuracy on a subset of the Extended Yale Face Database B.

*normalize error*	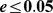	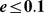	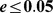	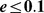
*illumination azimuth*	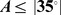	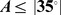	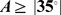	*A* 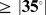
	and	and	or	or
*illumination elevation*	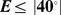	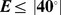	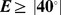	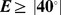
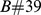	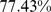	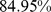	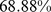	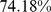
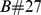	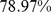	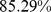	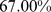	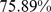
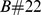	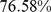	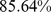	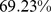	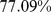
*Average Accuracy*	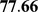 **%**	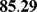 **%**	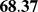 **%**	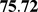 **%**

**Figure 9:**
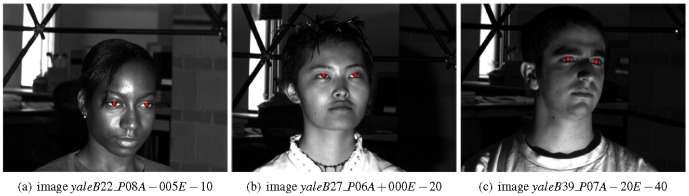
Some images of the Extended YALE database B in which the approach correctly detects the pupils. Reprinted from [29] under a CC BY license, with permission from Athinodoros S. Georghiades, original copyright 2001.

**Figure 10:**
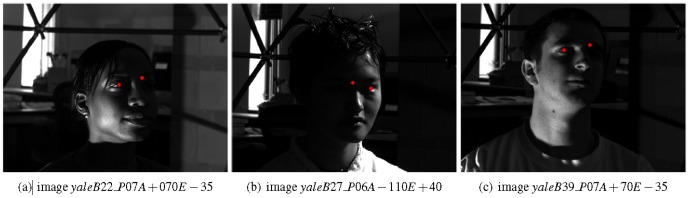
Some images of the Extended YALE database B in which the approach failed in the detection of the center of one or both eyes. Reprinted from [29] under a CC BY license, with permission from Athinodoros S. Georghiades, original copyright 2001.

By analyzing the results, it is possible to note that the proposed approach was able to deal with light source directions varying from 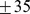 azimuth and from 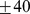 elevation with respect to the camera axis. The average accuracy obtained under these conditions was 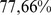 (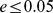) and 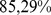
(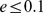). For higher angles, the method was often successful for the less illuminated eye and sporadically for the most illuminated one: if the eye was uniformly illuminated, the pupil was correctly located, even for low-intensity images. In figure 9, some images of the Extended YALE database B in which the approach correctly detected the pupils even under different lighting conditions and pose changing are shown. In figure 10, some images in which the detection of the pupils was either less accurate or completely failed are instead reported.

**Figure 11:**
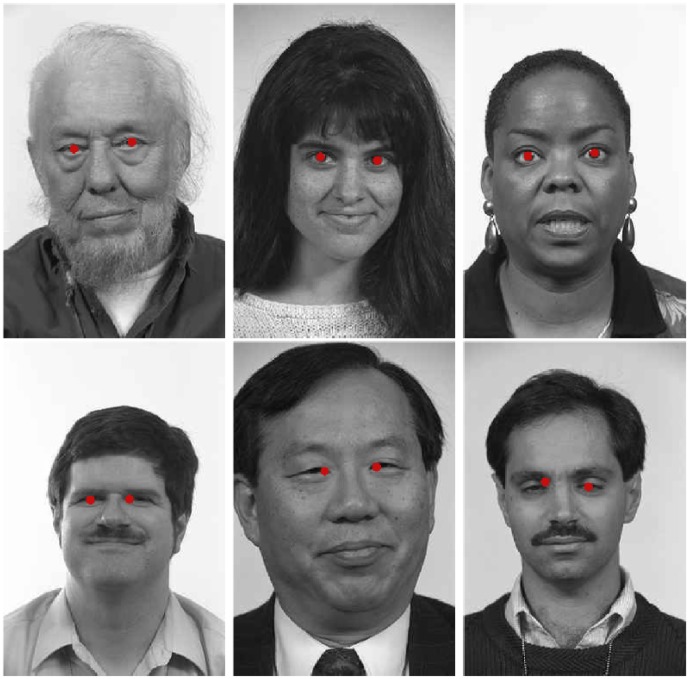
Some images of the FERET database and the relative correct (top) and wrong (bottom) pupil detection results obtained by the proposed approach. Reprinted from [30] under a CC BY license, with permission from Jonathon P. Phillips, original copyright 1998.

A final additional experiment was conducted on the color FERET database [30]. The color FERET database contains a total of 11,338 facial images collected by photographing 994 subjects at various angles over the course of 15 sessions between 1993 and 1996. The images in the color FERET Database are 512 by 768 pixels. In our case, we were only interested in the accuracy of the eye location in frontal images; therefore only the frontal face (fa) partition (994 images) of the database was considered. The results obtained were 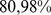 (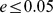) and 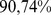
(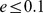) that are again comparable (sometimes outperform) with those approaches proposed in literature (that make use of training phase and machine learning strategies). This statement can be proven reporting some data relating to the results obtained by some methods in the literature on the same data-set. For example the method proposed in [12] performs 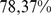 (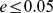) and 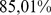 (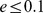), the method proposed in [3] performs 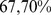 (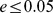) and 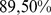 (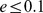), the method proposed in [25] performs instead 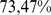 (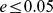) and 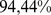 (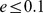). Figure 11 reports some images of the color FERET database and the relative correct pupil localization results (first row). The same figure (second row) also shows some images where the proposed pupil detection failed (due to partially closed eyes).

A final consideration should be made: during all the above experimental phases, no adjustment was made to the proposed method that, in light of its “unsupervised” nature, allows the users to change the operating environment while maintaining the detection capability of the centers of the eyes.

## Conclusions and Future Works

A new method to automatically locate the eyes, and in particular to precisely localize their centers (the pupils) in periocular images (even in presence of noise, challenging illumination conditions and low-resolution) has been proposed in this paper. Input image can be specifically acquired (i.e. close-up view of the eye for biometrics) or automatically cropped from facial image by means of one of the large number of face detectors in the literature. In the proposed solution, the pupil is localized by a two steps procedure: at first self-similarity information are extracted by considering the appearance variability of local regions and, then, they are combined with a shape analysis based on a differential analysis of image intensities. The proposed approach does not require any training phase or decision rules embedding some a priori knowledge about the operating environment. Experimental evidence of the effectiveness of the method was achieved on challenging benchmark datasets of facial images. The results obtained are comparable (sometimes outperform) with those obtained by the approaches proposed in literature (that make use of training phase and machine learning strategies).

With regard to the computational load, the calculation of the similarity space has a complexity 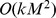, where 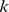 is the number of pixels in the image and 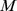 represents the maximal considered scale. The differential calculus is, in each considered scale, linear with the size of the image and then 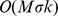. However, considering that the calculation of the two spaces is embarrassingly parallel (no effort is required to separate the problem into a number of parallel tasks) it is possible to approximate the computational load to the maximum of the two terms above. This therefore leads to a complexity comparable to that of the state of the art methods, however, offering better performance of detection and although not requiring training or other specific post-processing steps that limit their ability to work under various operating conditions.

To give a better idea of the real computational load of the algorithm, the average CPU time taken to process (working in a R2012a Matlab developing environment running, without parallel computing constructs, on a Sony VAIO PCG-71213w) the 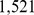 images of the BioID database (experiment #1 in section Experimental Results?) were measured. In particular, the proposed approach experienced about 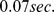 to detect facial regions, 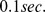 to compute self-similarity map and 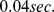 to compute the differential analysis of image intensities for each candidate eye region. Considering also the secondary operations (sum of the maps, search for the maximum, etc), overall, in the implemented version, the system is able to process about 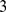 frames per second.

Future works will address the implementation in a intermediate-level language in order to speed-up the calculation. Where appropriate, processor supplementary instructions will also be used to achieve real-time processing. Moreover, a tracking algorithm will be integrated in order to suppress the sporadic experienced errors.
